# Genetic Basis of *Acinetobacter* sp. K1 Adaptation Mechanisms to Extreme Environmental Conditions

**DOI:** 10.3390/life13081728

**Published:** 2023-08-11

**Authors:** Nikola Petrová, Jana Kisková, Mariana Kolesárová, Peter Pristaš

**Affiliations:** 1 Department of Microbiology, Institute of Biology and Ecology, Faculty of Sciences, Pavol Jozef Safarik University in Kosice, Srobarova 2, 041 54 Kosice, Slovakia; nikola.siposova@student.upjs.sk (N.P.); jana.kiskova@upjs.sk (J.K.); mariana.kolesarova@upjs.sk (M.K.); 2 Centre of Biosciences, Institute of Animal Physiology, Slovak Academy of Sciences, Soltesovej 4-6, 040 01 Kosice, Slovakia

**Keywords:** *Acinetobacter* sp., resistance, plasmids, genome, brown mud

## Abstract

Anthropogenic pollution often leads to the generation of technosols, technogenic soils with inhospitable conditions for all living organisms including microbiota. Aluminum production near Ziar nad Hronom (Slovakia) resulted in the creation of a highly alkaline and heavy-metal-rich brown mud landfill, from which a bacterial strain of a likely new species of the genus *Acinetobacter*, *Acinetobacter* sp. K1, was isolated. The whole-genome sequence analysis of this strain confirmed the presence of operon units enabling tolerance to the heavy metals copper, zinc, cobalt, cadmium, chromium, and metalloid arsenic, which are functionally active. Despite the predominance of plasmid-related sequences in the K1 genome, the results indicate that most of the resistance genes are chromosomally encoded. No significant alkali tolerance of *Acinetobacter* sp. K1 was observed in vitro, suggesting that community level mechanisms are responsible for the survival of this strain in the highly alkaline, brown mud bacterial community.

## 1. Introduction

Bacteria belonging to the genus *Acinetobacter* are characterized by the ability to inhabit a wide range of environments and to adapt to the prevailing extreme conditions. Many strains of *Acinetobacter* spp. can tolerate increased concentrations of heavy metals and different pH values of the environment and resist the action of various types of antibiotics [1,2]. The high tolerance is possible due to the activity of several protein groups encoded by genes located mainly on mobile genetic elements and thus can be transmitted horizontally within the bacterial population. In addition, bacteria of the *Acinetobacter* genus are able to form capsules, increasing their resistance to environmental impacts even more [3]. Environmental *Acinetobacter* spp. species, including *A. lwoffii* strains, are generally considered non-pathogenic compared to clinical *A. baumannii* strains. On the other hand, in recent years, the number of infections caused by this bacterial species has noticeably increased [4,5,6,7,8].

Various industrial activities cause environmental pollution with a subsequent negative impact on human and environmental health. The generation of large quantities of technosols or technogenic soils with diverse toxic properties is one of the emerging phenomena of these activities [9,10,11]. Such waste sludges present a challenge in finding effective methods for their removal. In recent years, research has shown the high potential of *Acinetobacter* spp. in biotechnologies and bioremediation of industrial waste sludges [12]. These approaches are being intensively studied because of the advantages offered by bacterial biomass, such as the short generation time of bacteria or the ability of their eventual genetic modification [13]. In recent decades, whole-genome sequencing has become a widely used method to better understand the organization of genes responsible for bacterial adaptation to polluted industrial environments.

The bacterial isolate *Acinetobacter* sp. K1 was isolated from a highly alkaline, brown mud landfill generated during the years of aluminum production near Ziar nad Hronom (Slovakia); the technosol was contaminated by high concentrations of heavy metals, including mercury [14,15]. The population in the brown mud was dominated by Gram-positive bacteria (*Bacillus*, *Micrococcus*, *Isoptericola* and *Arthrobacter*) and acinetobacters were the only representative of Gram-negative bacteria [14]. As acinetobacters are reported to prefer a slightly acid pH as the optimum for growth [16], we focused on the determination of genes responsible for the adaptation of the K1 isolate to extreme conditions of the brown mud and the putative role of horizontal gene transfer in the adaptation as the presence of multiple DNA plasmids (with a size of 3 kbp–25 kbp) were detected in the K1 genome [17].

## 2. Materials and Methods

### 2.1. Origin of the Bacterial Isolate Acinetobacter sp. K1 and Growth Conditions

The bacterial strain *Acinetobacter* sp. K1 was isolated from a brown mud created during aluminum production of the Slovalco Co. near Ziar nad Hronom (48°35′3″ N, 18°51′39″ E) in our previous research. To a 0.5 g solid brown mud sample, 10 mL of a sterile phosphate-buffered saline solution was added and the mixture was intensively mixed for 20 min. Subsequently, the aliquots were spread on tryptone soya agar (TSA) (Oxoid, Columbia, MD, USA) and cultivated for 24–72 h at 22 °C [14]. After the cell and colony morphology examination, subculturing and the purity check, the K1 strain was preliminary identified using matrix-assisted laser desorption ionization time of flight mass spectroscopy (MALDI-TOF MS) and phylogenetic analysis based on the 16S rRNA gene sequence. The phenotypic pattern was analyzed using a GEN III MicroPlate system (Biolog, Hayward, CA, USA) [14]. The isolate was routinely cultivated on TSA (BD Difco, Sparks, MD, USA) or liquid Luria–Bertani (LB) medium (BD Difco, Sparks, MD, USA) at 25 °C for 12–16 h in all experiments. 

### 2.2. DNA Extraction and Purification

The genomic DNA was extracted from an overnight LB bacterial culture using an E.Z.N.A.^®^ Bacterial DNA Kit (Omega Bio-tek, Norcross, GA, USA) according to the manufacturer’s instructions. Subsequently, the genomic DNA was purified by ethanol precipitation. We added 0.1 volumes of 3 mM sodium acetate (pH = 6) and 2.5 volumes of 96% ethanol to the DNA, mixed by inverting several times and incubated for 10 min at −70 °C. After 30 min of centrifugation at 4 °C and 13,000× *g*, we removed the supernatant and added 500 µL of 70% ethanol to the DNA sediment. The DNA was centrifuged for 15 min at 25 °C, the supernatant was removed, and the dried DNA was dissolved in a 10 mM TRIS solution (pH = 8) (Applichem, Germany). The electrophoresis was performed in a 1% agarose gel stained with ethidium bromide (0.5 μg/L) under the following conditions: 5 V/cm at 25° C for 30 min. The DNA was visualized under UV light using a Gel Logic 212 PRO Imaging System (Carestream, Health Inc., Rochester, NY, USA). The optimal purity and concentration were determined spectrophotometrically using a NanoDrop 2000c Spectrophotometer (Thermo Scientific, Carlsbad, CA, USA).

### 2.3. The Whole-Genome Sequence Analysis

Whole-genome sequencing of the isolate *Acinetobacter* sp. K1 was carried out using an Illumina HiSeq 2000 platform and the paired-end strategy (2 × 150 bp) in the NovaSeq 6000 mode using an S2 PE150 XP kit (Eurofins Genomics Europe Sequencing GmbH, Köln, Germany). The obtained sequences were processed using the tools implemented in the Unipro UGENE v35.0 bioinformatics software [18,19]. The quality of the raw data was evaluated by the program FastQC v0.11.8. Reads with low quality scores (<20) were removed using Trimmomatic v0.38 software. The resulting sequences were assembled de novo with the program SPAdes 3.12.0. Subsequently, the sequences shorter than 200 bp were excluded from further analyses. The contigs obtained were checked against the GenBank database available at https://blast.ncbi.nlm.nih.gov/Blast.cgi (accessed on 1 April 2022) using the Blastn algorithm [20] and Rapid Annotation using Subsystem Technology (RAST) was used to determine the gene contents of the *Acinetobacter* sp. K1 genome based on functional subsystem classifications [21,22,23].

### 2.4. Taxogenomics

We used the Type (Strain) Genome Server (TYGS, https://tygs.dsmz.de/) (accessed on 25 July 2023) for the taxogenomic comparisons of the K1 genome based on the Genome BLAST Distance Phylogeny (GBDP) method and comparison of the 16S rRNA gene [24]. The K1 genome relatedness to the reference genomes of *A. lwoffii* strains (accession numbers are listed in Table S2) was calculated using The Kostas Lab Average Nucleotide Identity calculator (ANI, http://enve-omics.ce.gatech.edu/ani/index) (accessed on 16 March 2023) with a cut-off value of 95% indicating the same species [25,26]. The similarity matrix created from the ANI values was used to build the phylogenetic tree using the Unweighted Pair Group Method with Arithmetic Mean (UPGMA) and visualized using the Iroki online tool available at https://www.iroki.net/viewer (accessed on 21 March 2023) [27].

### 2.5. Identification of the Plasmid-Related Sequences and Localization of Determinants Important for the Adaptation Mechanisms of the K1 Isolate

The putative plasmid-related sequences identified using the RAST server were verified using the Conserved Domain Database (CDD) [28] and ORF finder (https://www.ncbi.nlm.nih.gov/orffinder/) (accessed on 5 December 2022). We focused on the presence of genes involved in resistance to extreme environmental conditions and especially on the genes located in plasmid DNA. In addition, we compared the contig sequences with the genomes in the GenBank database using the Blastn and Blastx algorithms [20]. Plasmid-related sequences were predicted using the mlplasmids v2.1.0 online server (https://sarredondo.shinyapps.io/mlplasmids/) (accessed on 9 May 2023) [29]. The average GC content of contigs was calculated using the Genomics %G~C Content Calculator available at https://www.sciencebuddies.org/science-fair-projects/references/genomics-g-c-content-calculator (accessed on 27 March 2023).

## 3. Results

### 3.1. The Whole-Genome Sequence Analysis and Taxogenomics of the K1 Isolate

The electrophoretic analysis of the total DNA of Acinetobacter sp. K1 showed the presence of multiple extrachromosomal DNA bands [17]. To evaluate the potential role of plasmid-encoded genes in adaptation of the isolate to extreme alkalic conditions, whole-genome sequencing of the isolate *Acinetobacter* sp. K1 was performed. The sequencing provided 6,158,720 reads of 1,847,616,000 nucleotides with 543× coverage of the K1 genome. The raw reads were assembled into a draft genome of 3,357,632 bp, organized into 94 contigs with N50 = 93,093 bp and L50 = 11. The genome was deposited into the GenBank database under accession number NZ_JALGQY000000000.1. Annotation by the RAST server led to the identification of 3413 protein-coding genes and RNA genes. No CRISPR-cas9 system gene(s) were detected in the K1 genome. A quality check by the RAST server did not identify any warnings or fatal problems. The general characteristics of the K1 genome were compared to those of twelve genomes of *A. lwoffii* strains, one *A. pseudolwoffii* strain and three *A. idrijaensis* strains available in the GenBank database using the RAST server (Table S1). The average GC content of the K1 genome (42.8%) was similar to all *Acinetobacter* spp. strains included in this analysis. The *A. idrijaensis* MII strain showed the highest number of protein-coding sequences. The number of protein-coding sequences in the K1 genome was the fourth highest among all analyzed strains.

**Figure 1:**
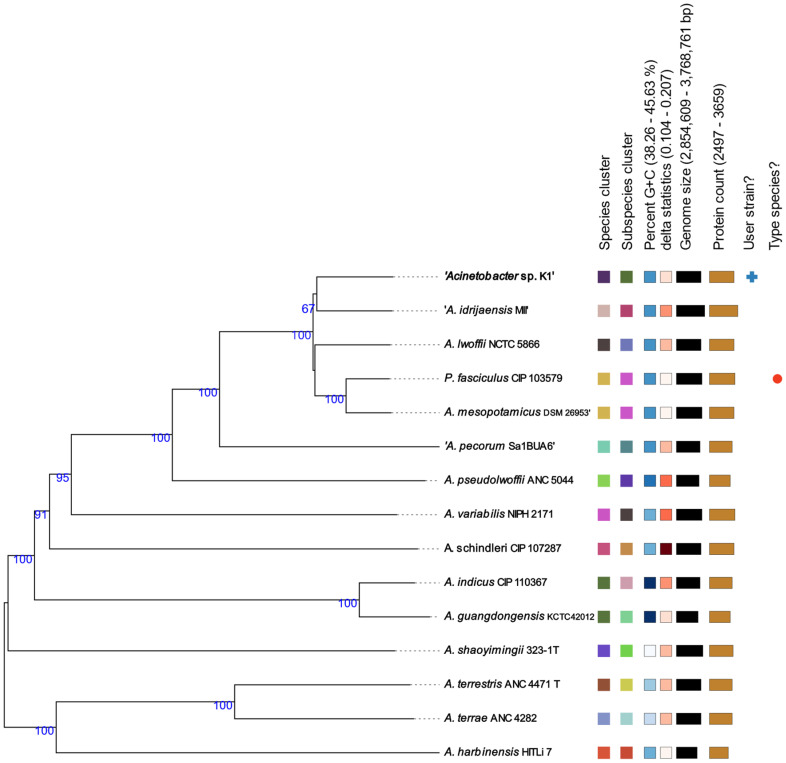
The GBDP phylogenetic analysis based on the whole-genome sequence comparison of different species of the genus Acinetobacter using the TYGS server [24]. Tree inferred with FastME 2.1.6.1 [30] from GBDP distances calculated from genome sequences. The branch lengths are scaled in terms of GBDP distance formula d5. The numbers above branches are GBDP pseudo-bootstrap support values > 60% from 100 replications, with an average branch support of 91.9%. The tree was rooted at the midpoint [31]. Leaf labels are annotated by affiliation to dDDH (digital DNA:DNA hybridization) species and subspecies clusters, genomic G+C content, delta values, overall genome sequence length and number of proteins. Delta statistics permit assessment of accuracy in terms of tree-likeness; the lower the delta value, the greater the accuracy [32].

The K1 isolate showed the closest relationship to the *A. idrijaensis* MII according to the GBDP phylogenetic analysis (Figure 1) with a dDDH value below 70%. This finding was also confirmed by the phylogenetic analysis of the 16S rRNA gene sequences (Figure S1). However, the bootstrap value was too low to reliably assign the K1 isolate to the *A. idrijaensis* species and the TYGS server predicted that the K1 strain is likely to be a new species of the *Acinetobacter* genus.

The ANI value between the K1 genome and the type strain of *A. lwoffii* was 95.84%. The highest ANI value of 96.26% was observed between the genomes of *Acinetobacter* sp. K1 and *A. lwoffii* NIPH478. A similarity of 96% or more was also shown with two other strains of *A. lwoffii* (SH145 and UBA2051). An ANI value higher than 95% was observed with *A. idrijaensis* MII, *A. mesopotamicus* DSM 26953, and *Prolinoborus fasciculus* CIP 103579. An ANI index lower than 90% was shown with the strains *A. lwoffii* CIP64.10, *A. lwoffii* F78 and *A. lwoffii* WJ10621 (Table S3). The dendrogram constructed based on the ANI similarity matrix (Table S3) grouped the *A. lwoffii* strains in several separate branches, indicating non-homogeneity (resp. high genetic variability) of the *A. lwoffii* species (Figure S2). The K1 isolate formed a separate group with *A. lwoffii* NIPH478 that was distant from the *A. idrijaensis* MII strain. 

### 3.2. Identification of the Plasmid-Related Sequences in the K1 Genome

Thirty-seven contigs (with a total size of 476,260 bp), representing 13.13% of the whole K1 genome, were predicted to be plasmid-related according to the mlplasmids v2.1.0 online server (Table S4). Three complete plasmid sequences were identified, including the sequence of the cryptic pALK1 plasmid (NZ_JALGQY010000067.1), whose sequence was obtained using the recombinant DNA techniques in our previous study (data not shown) and two other small plasmids unnamed1 (NZ_JALGQY010000057.1) and unnamed2 (NZ_JALGQY010000058.1) (Table S5). Plasmid unnamed1 carries the gene for a yet undescribed replication protein rep_pAB02_ORF2. This gene is often located near the *rep*M gene; however, in plasmid unnamed 1, the presence of the genes encoding the Replicase superfamily and PriCT_1 protein of the Primase superfamily were detected behind the rep_pAB02_ORF2 gene. This plasmid also encodes a protein of the Serine recombinase family and the transcriptional regulator HipB. Plasmid unnamed2 encodes the replication protein Rep_3/RepM, the mobilization protein MobA_MobL and the relaxase Tra_Ti. In addition, the PhdYeFM superfamily of antitoxins and the YoeB-like toxin of the type II toxin–antitoxin system are encoded by the plasmid unnamed2 sequence. Several other contig sequences showed high similarities to *Acinetobacter* spp. plasmid sequences using the Blastn algorithm. However, some of these contigs (e.g., NODE 34, 50, 56 and 261) were proposed as being of chromosomal origin according to the mlplasmids v2.1.0 online server. The average GC content showed that majority of plasmid-related sequences clearly differed from the chromosomal DNA of the K1 isolate, indicating that plasmids were probably obtained by horizontal gene transfer events (Tables S4 and S5).

The most frequently occurring genes encoding replication proteins of putative plasmid-related sequences (such as NODE 38, 43, 47, 55, 66, 223 and plasmids pALK1, unnamed1 and unnamed 2) are *rep_*3, *rep*M and *rep*_pAB02_ORF2 (Table S5). The mobilization protein MobA_MobL is encoded by a gene located on plasmid unnamed2 and on contigs NODE 55 and 2715, while genes coding proteins MobM and MobC were detected only on plasmid pALK1 and on contig NODE 43, respectively. The sequences of plasmid unnamed2 and contigs NODE 55 and 2568 encode the Tra_Ti protein. A considerable number of the analyzed contigs contain conserved domain sequences of the IS3, IS5_2, IS5_3, IS21, IS481 or ISNCY_2 Transposase families. Conserved domain sequences of integrases, for example, XerC, were also found in the K1 genome. The genes coding the ComFC protein superfamily were detected within contig NODE 47. Conserved “competence domain” sequence of ComEC proteins was located on contig NODE 97. In addition, contig NODE 97 also encodes transposases and, according to all predictions, it is probably a plasmid-related sequence.

### 3.3. Genetic Determinants Important to Adaptation Mechanisms of the K1 Isolate

**Figure 2:**
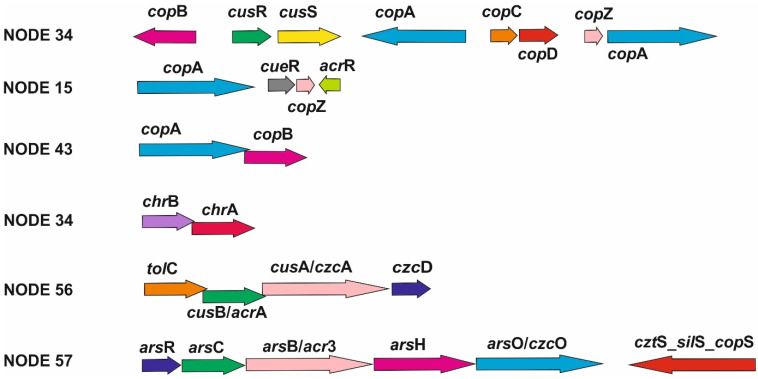
Organization of identified operon units mediating tolerance to heavy metals and metalloids in the strain *Acinetobacter* sp. K1.

Multiple genes involved in heavy-metal-resistance mechanisms were identified on contig NODE 34, e.g., genes of the *cop* operon (*cop*ABCDZ, *cus*SR and *pco*B) that are responsible for copper resistance; *czc*D, *chr*A and *chr*B which are involved in resistance mechanisms to high concentrations of cadmium, zinc, cobalt and chromium; *znt*A encoding a P-type ATPase that participates in the transport of zinc, cadmium and lead; and genes encoding the DmeF protein of the CDF protein family of efflux transporters. We have not been able to reliably determine the origin of the DNA due to the different results of the analyses (Figure 2, Tables S4 and S5). The mlplasmids v2.1.0 server determined this contig as chromosomal DNA; on the other hand, it showed a low average GC content and consists of the genes coding the IS6 family of transposases and a serine recombinase. The genes encoding the CopA and CopB proteins were also found on contig NODE 43, which was identified as plasmid DNA with a high probability. It carries genes encoding the replication protein RepM, the mobilization protein MobC and proteins of the IS48 and IS3 transposase families.

Heavy-metal resistance or efflux pump genes (*czc*A, *czc*D, *tol*C, *arc*A, *cad*R-*pbr*R, *znt*A and *zit*B) were also detected on other contigs, e.g., NODE 56 (Figure 2, Table S5), NODE 130, NODE 1947 (Table 1) and NODE 261 (Table S5). The mlplasmids server determined NODE 130 and NODE 1947 as plasmid-related DNA (Table S4). However, replication-protein-encoding genes have not been identified on any of these contigs. The conserved domain sequences of the IS6 and IS240 transposase families were found only on contig NODE 130. Contig NODE 261 showed a 99.37% similarity (100% coverage) to *A. lwoffii* FDAARGOS_557 plasmid DNA (CP054804.1) according to the Blastn analysis; however, the mlplasmids v2.1.0 server evaluated this sequence as being of chromosomal origin (Tables S4 and S5). In addition, this contig encodes FeoA and FeoB proteins involved in the transport of iron ions into the interior of bacterial cells [33].

Arsenic-resistance determinants of *ars* and *smt* operon were found on several contigs of putative chromosomal (NODE 15 and NODE 50) and plasmid origin (NODE 29, 48 and 57). However, only in the case of contig NODE 15 have we been able to reliably determine the chromosomal origin of the DNA. Contig NODE 50 showed significant similarities with plasmid pALWED3.1 (CP083572.1) using Blastn analysis and a lower average GC content. On the other hand, the mlplasmids v2.1.0 server determined this contig to be a sequence of chromosomal origin. The sequence of contig NODE 57 encodes genes for ArsH, ArsC, ArsO, ArsR and Acr3 proteins (Figure 2). Even though this sequence showed the highest similarity to Acinetobacter chromosomal DNA, the mlplasmids v2.1.0 server classified it as a plasmid-related sequence (Table 1).

Several other plasmid-encoded genes in the K1 genome were detected, e.g., *crc*B (NODE 1680) encoding a protein important for the maintaining fluoride concentrations inside bacterial cells [34]; *tel*A involved in the resistance to tellurite (NODE 2568); a conserved domain sequence of *ter*C encoding the integral membrane protein superfamily important for tellurium efflux from bacterial cells; and a gene encoding the Phenol_MetA_deg superfamily (NODE 211) involved in the MetA pathway of phenol degradation (Table 1). 

**Figure 3:**
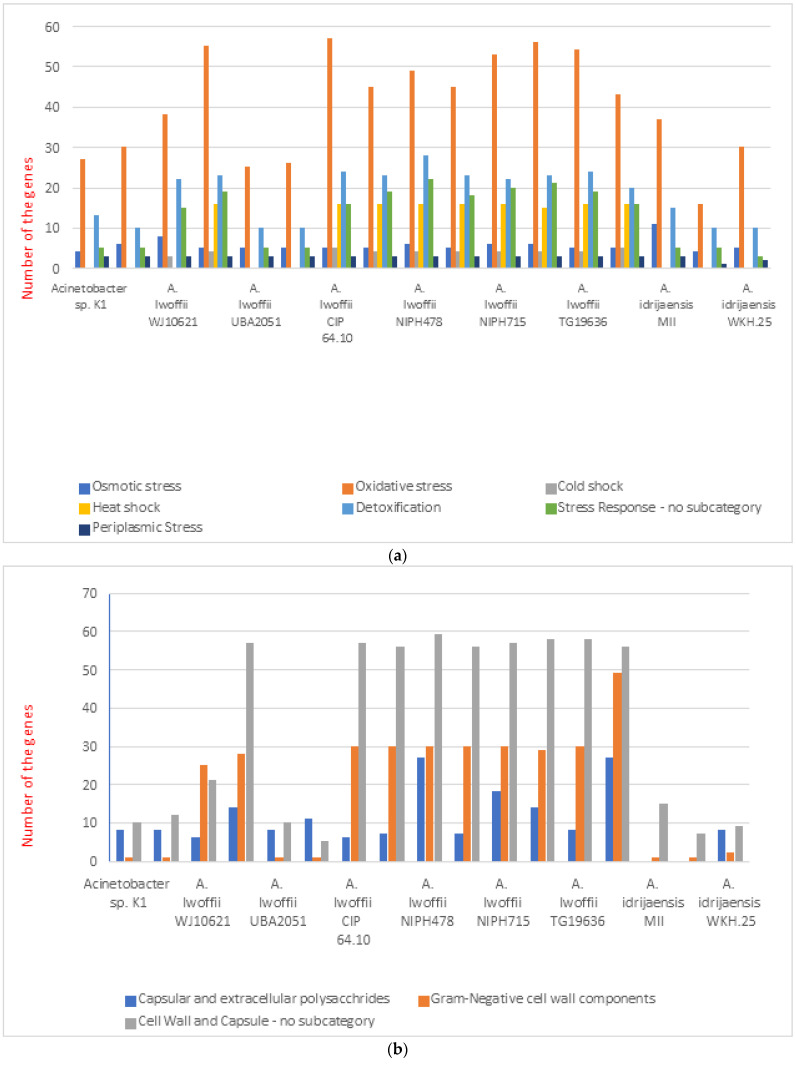
Comparison of the number of stress response genes (**a**) and genes involved in cell wall and capsule biosynthesis (**b**) in *A. lwoffii* strains using the RAST server [21,22,23].

The bacterial strain *Acinetobacter* sp. K1 carries the genes that are important for adaptation to alkaline pH, e.g., Na^+^/K^+^ antiporter genes, genes for cytochrome *bd* and genes for stress responses and production of cell wall components and bacterial capsules (Figure 3).

*Acinetobacter* sp. K1 also harbors genes important for the intracellular regulation of osmotic pressure. First, we detected the genes *kup*, *msc*L and *kef*A (*msc*S) encoding transport proteins that play a role in transport and subsequent accumulation of potassium cations [35] and/or organic solutes [36] in the bacterial cytoplasm and a conserved domain of ion transport protein family pfam00520. The K1 genome also encodes genes responsible for the synthesis of the compatible organic solutes betaine (*bet*A, *bet*B), proline (*pro*A, *pro*B, *pro*C), glutamate (*gdh*A, *gln*A, *glt*B, *glt*D) and trehalose (*ots*A, *ots*B). Only genes encoding Kup, pfam00520, OtsA and OtsB proteins are plasmid-encoded (Table 1).

In the K1 genome, we found chromosomally encoded genes that are important for dealing with oxidative stress caused by various negative environmental conditions (such as high concentrations of some heavy metals [37]): *sod*B and *sod*C encoding superoxide dismutases, *ahp*C encoding alkylhydroperoxidase, *osm*C encoding organic hydroperoxide reductase, *gsh*A encoding glutamate-cysteine ligase and *gsh*B encoding glutathione synthetase.

## 4. Discussion

### 4.1. Taxogenomic Placement of the K1 Isolate

The bacterial isolate *Acinetobacter* sp. K1 was sampled from an environment characterized by extreme alkaline pH (11.6) and contamination with heavy metals (Hg: 10 mg/kg, Cu: 220 mg/kg, Cr: 400 mg/kg, V: 700 mg/kg, Pb: 150 mg/kg and As: 800 mg/kg) [14,15]. The initial identification of the K1 strain was carried out using MALDI-TOF MS by Kopčáková et al. [14]. The strain was classified as *A. lwoffii* based on the protein profile with a MALDI score of 1.961, which is below the value of 2 that is considered reliable for identification at the species level. The 16S rRNA gene sequence analysis also confirmed the highest similarity (99% identity, 96% coverage) of the K1 isolate to *A. lwoffii* DSM 2403 (NR 026209.1) [14]. 

In 1986, Bouvet and Grimont established the basics for the species classification of bacteria belonging to the genus *Acinetobacter* using DNA–DNA hybridization and comprehensive phenotypic testing [38]. In this study, we tried to reevaluate the classification of the K1 isolate as the *A. lwoffii* species using different phylogenetic approaches with different results. The K1 strain was identified as a potential new species based on the TYGS server analysis; however, it showed the highest similarity to the *A. idrijaensis* MII strain. A phylogenetic analysis based on the ANI similarity matrix showed that the closest relationship of the K1 strain was to the *A. lwoffii* NIPH478. However, Campos-Guillén et al. [39] demonstrated that the 16S rRNA gene of the *A. idrijaensis* MII strain is 99% identical to the *A. lwoffii* species, while the same being true for the K1 isolate. In addition, the taxonomic name *idrijaensis* was not published according to the rules of the International Code of Bacterial Nomenclature (Bacteriological Code) [40]. Similarly, Nemec [41] refuted the classification of the *Acinetobacter* strain GC2 as the species *Acinetobacter mesopotamicus*, which was proposed by Acer et al. [42]. Finally, Nemec [41] assigned this strain to the species *A. lwoffii* based on the ANI values and DNA–DNA hybridization. Because of these discrepancies, further analyses would be necessary for more precise species identification of these strains.

### 4.2. Genetic Determinants Important for Adaptation of the K1 Strain to Extreme Environmental Conditions

Various studies demonstrated the tolerance of bacterial strains belonging to the *Acinetobacter* spp. to heavy metals and their ability to survive in diverse environments [1,2,43,44,45]. The ability of the strain *Acinetobacter* sp. K1 to tolerate higher concentrations of heavy metals and an alkaline pH was confirmed experimentally in our previous work [17]. Our preliminary analyses also identified the complex plasmid population in the K1 isolate [17]. It has already been shown that plasmids could play a key role in the adaptability of *Acinetobacter* to their living conditions, including heavy metal resistance [46]. Moreover, some plasmids in *Acinetobacter* potentiate the accumulation and horizontal transfer of diverse accessory genes [47].

Bacterial genes involved in heavy-metal-resistance mechanisms are often organized into characteristic operon units. The *cop* operon is the best-studied operon unit, conferring bacterial tolerance to high copper concentrations [48,49]. This operon was detected on contig NODE 34 that we could not reliably identify as plasmid or chromosomal DNA using the various bioinformatic analyses. The discrepancy could be due to the mlplasmids v2.1.2 server being optimized for searching plasmid sequences in the *A. baumannii* species complex. Nevertheless, this contig encodes all the genes necessary to exclude copper ions from the cell (Figure 2). The presence of *cus*RS regulatory genes, as well as prediction by the mlplasmids v2.1.2 server, point to a chromosomal origin of this sequence [49]. In addition, the *chr* operon was found on this contig. The ChrA protein enables chromate efflux from the cell and the ChrB protein is a regulatory protein that responds to the concentration of chromium in the intracellular environment [50]. Such an arrangement of genes is characteristic for *Acinetobacter* spp. [50], but in various *Acinetobacter* species, we can also observe the presence of other genes that complement this operon [51]. Mindlin et al. [50] identified a recombination site XerC/XerD near this operon. We detected the conserved domain of the IS6 family of transposases in the contig sequence. Despite the likely chromosomal origin of this sequence, the presence of this enzyme suggests the possibility of horizontal gene transfer through transposition. Contig NODE 43 encoding the genes *cop*A and *cop*B is probably part of the plasmid DNA of the K1 genome. Genes involved in the resistance to copper are also present on probably chromosomal contig NODE 15. We can assume that the copper tolerance of the K1 strain determined in our previous study [17] is probably mediated by the *cop* operon genes distributed mainly within the chromosomal DNA of the K1 isolate.

Bacterial resistance to cobalt, zinc and cadmium is provided by the *czc* operon encoding the CzcABC proteins [52] that form an efflux pump. The genes coding CusB/AcrA and TolC protein were detected on contig NODE 56. Protein CusB/AcrA could function as a fusion periplasmic protein CzcB; TolC protein is an outer membrane protein like CzcC. These components, together with the inner membrane protein CzcA, can form a complex efflux pump of the CzcABC type and thus ensure the exclusion of Co^2+^, Zn^2+^ and Ca^2+^ ions from the cell, supported by the efflux pump CzcD, which is encoded by several contigs of chromosomal and plasmid origin. The tolerance of the K1 strain to cobalt and zinc (MIC 1 mM for cobalt and 2 mM for zinc) [17] is probably the result of cooperation of these determinants. However, the determinants responsible for HGT were only detected within contig NODE 34. Similar operons with an identical arrangement of *czc*ABC genes (Figure 2) occur in various species of bacteria [53,54,55].

Operon *ars* (the components encoded by NODE 50 and 57) ensures the resistance to arsenic in various bacterial species [56]. Contig NODE 57 encodes the ArsC protein that catalyzes the reduction of arsenate to arsenite and the ArsH protein that reduces organoarsenical compounds. Arsenite can be excluded from the bacterial cell by the Acr3 protein performing the function of the typical *ars* operon protein, ArsB. Regulation of the operon unit is provided by the ArsR protein. The activity of the operon is supported by the protein ArsO, whose function is not yet precisely defined [57]. The cztS_silS_copS protein family contains a domain characteristic for bacterial signal proteins and is associated with the efflux systems of heavy metals such as copper, silver, cadmium and zinc [58,59,60]. Contig NODE 50 contains genes coding the three primary components that are necessary for the detoxification and elimination of arsenite in the bacterial cells. It is not clear whether the NODE 50 and 57 contigs are of plasmid origin; however, they carry genes encoding transposases. The tolerance of *Acinetobacter* sp. K1 to arsenic was examined in our previous study and the MIC of arsenic was determined as a value of 2.6 mM [61]. 

In the K1 genome, we failed to detect the complete *mer* operon, which plays an important role in mercury resistance. The genome encodes only the regulatory protein of this operon, MerR. According to our previous research, the MIC of mercury of the K1 strain was established as 0.025 mM [61]. Despite the absence of important genes of the *mer* operon, this isolate survived in the sewage sludge contaminated with mercury, in addition to other heavy metals. Our hypothesis for this phenomenon is that the K1 isolate survived thanks to the detoxification mechanisms of the bacterial community presented in the brown mud, enabling the survival of sensitive strains. Further studies will be necessary to better understand these findings. 

Several studies documented that various *Acinetobacter* strains are able to successfully thrive in acidic or alkaline environments [62,63,64]. According to our previous study, the bacterial strain *Acinetobacter* sp. K1 was able to grow at a maximum pH of 9 in vitro [17]. Bacteria use various mechanisms to maintain the pH homeostasis of their intracellular environment. These mechanisms include, for example, increased expression of cation/proton antiporters [65,66]. In the K1 genome, Na^+^ and K^+^ ion antiporters were detected, such as the pfam07885 and pfam00520 protein families, located on contig NODE 57. In addition, the subunits A, B, C, D, E, F and G of the Na^+^/H^+^ antiporter were detected in the K1 genome. Some bacteria (*E. coli*) use an increased expression of cytochrome *bd* (Cyd) as a protective mechanism [67]. The gene coding cytochrome *bd* is encoded by contig NODE 34 in the K1 genome. Additionally, we compared the number of genes for stress responses among the strains of *A. lwoffii*, *A. pseudolwoffii* and *A. idrijaensis* (Figure 3a). The highest similarity was observed with environmental strains *A. lwoffii* ZS207, *A. lwoffii* UBA 2051, *A. lwoffii* UBA1649 and *A. idrijaensis* WKH.25 which have different origins (Table S1), but these strains are characterized by a lower number of genes of this group. Modification of the bacterial cell wall and the production of secondary polymers of the cell wall (teichuronopeptide and SlpA protein) are other possible mechanisms of the protection against high alkalinity [65,66]. *Acinetobacter* spp. synthesize bacterial capsules, which are composed of tightly arranged repeating units of polysaccharides. Production of this protective cover is primarily a defense mechanism of clinically significant types of bacteria, creating a barrier around the cell wall, thus providing protection against adverse environmental conditions (disinfection, drying, host immune response, etc.). They even confer resistance to various clinically relevant antimicrobial agents [3]. We observed a significantly lower number of genes involved in the formation of the cell wall components and capsules in the K1 genome compared to other *A. lwoffii* strains (Figure 3b). The highest similarity between gene numbers was observed between the same strains as in the case of genes involved in for stress responses. In addition, combinations of available protective mechanisms enable the survival of this strain up to a pH of 9 in vitro. One of the explanations for this observation could be that a highly alkaline environment of brown mud is probably inhabited by other alkali-tolerant microorganisms, providing protection to more sensitive bacterial strains, including the isolate *Acinetobacter* sp. K1. Bacterial survival based on their cooperation is also supported by biochemical tests performed using GEN III MicroPlate™ (Biolog, Hayward, CA, USA). The bacterial strain K1 is a non-fermenting strain that uses simple carboxylic acids as a carbon source instead of simple carbohydrates [14].

High osmotic pressure environments cause lethal turgor reduction and cytoplasm dehydration in bacteria. However, in microorganisms, two main mechanisms to manage osmotic pressure are observed: K^+^ accumulation in the cytoplasm and the biosynthesis and subsequent accumulation of various compatible organic solutes [68]. In a comparative genomic analysis of 312 *Acinetobacter* spp. strains [69], genes encoding the Kup system of potassium transport with low affinity [35] were found in all analyzed strains. While most of the analyzed strains have 2-4 potassium transport systems such as Kup, Ktr, Trk, Kdp and Ktr [69], the bacterial strain *Acinetobacter* sp. K1 harbors mechanosensitive channels MscL and MscS [70,71,72] and a conserved domain of the ion transport protein family pfam00520 in addition to the Kup system. The K1 strain can produce the compatible organic solutes betaine, trehalose, proline and glutamate which are not only involved in maintaining the bacterial cell osmotic balance but also in the stabilization of the cellular components including proteins during the high ionic stress [73].

In some cases, extreme environmental conditions lead to the production of reactive oxygen species, and massive accumulation in the bacterial cells is lethal [74]. *Acinetobacter* spp. were found to encode superoxide dismutases SodB or SodC [69,75] that catalyze the dismutation of O**_2_^•^^−^** to O**_2_** and H_2_O_2_ [76], which we also detected in the K1 strain. Bacterial strains of the genus *Acinetobacter* often encode enzymes catalyzing the conversion of H_2_O_2_ (catalases KatE and KatG, and alkylhydroperoxidase AhpC) and the repair of oxidized cysteine and methionine residues in bacterial proteins (disulfide isomerase DsbC and methionine sulfoxide reductase Msr) [69]. Bioinformatic analyses of the K1 genome confirmed only presence of the *ahp*C gene supplemented by the genes for organic hydroperoxide reductase (*osm*C/*ohr*) [77], glutamate-cysteine ligase (*gsh*A) and glutathione synthetase (*gsh*B) which can also protect bacteria against oxidative stress [78].

In addition to the proteins important for plasmid DNA replication, mobilization and transposition, we also detected genes of the competence system (*pil*A, *pil*Q, *pil*C, *pil*B, *pil*D, *pil*Dm, *pil*T, *pil*M, *pil*N, *pil*O, *pil*P, *pil*Y1, *pil*V, *pil*W, *pil*R, *fim*T and *fim*B) in the K1 genome, located in several contigs, which are important in the formation of the type IV pilus (T4P) [79]. We also detected the genes encoding proteins ComEC and ComFC, which are important for transport of single-stranded DNA across the inner membrane of the bacteria during the natural transformation process [79]. In conclusion, genetic determinants found in the K1 genome provide various possibilities for HGT, including conjugation, transposition and transformation. On the other hand, despite the frequent presence of plasmid DNA in the K1 genome, the genes important for various tolerance mechanisms to heavy metals tend to be encoded by the chromosomal DNA of this strain.

## 5. Conclusions

The bacterial strain *Acinetobacter* sp. K1 was isolated from alkaline and heavy=metal-contaminated technogenic soil produced by industrial activities near Ziar nad Hronom (Slovakia). The phylogenetic analyses suggest that this strain is potentially a new species of the *Acinetobacter* genus. A whole-genome analysis was used to decipher the molecular basis of the K1 strain’s survival in its extreme environment. The analysis confirmed the presence of the complete operon units for *cop*, *czc*, *chr* and *ars*, providing resistance to copper, zinc, cobalt, cadmium, chromium and arsenic. The lack of mercury resistance determinants in the K1 genome is probably the result of the presence of the complex bacterial community in the brown mud, in which the resistance mechanisms of other microorganisms provide protection to more sensitive bacterial strains. According to our analyses, despite the presence of multiple plasmids in the K1 genome, the genes responsible for the adaptation to extreme environmental conditions are mainly chromosomally encoded. Nevertheless, we detected other important genes potentially mediating horizontal gene transfer of resistance genes between the bacterial populations of the brown mud.

## Figures and Tables

**Table 1:** Plasmid-related sequences of the K1 genome encoding genes important for adaptation of *Acinetibacter* sp. K1 to high concentrations and/or degradation of heavy metals, metalloids, phenol and antibiotics according to the mlplasmids v2.1.2 server [29].

Contig	Size (bp)	GenBank Accession No.	Replication	HGT	Resistance
NODE 4-2	34,994	NZ_JALGQY010000005	-	IS5_3 transposase superfamily	AcrA, AcrB; RND_mfp superfamily
NODE 13	78,976	NZ_JALGQY010000014	-	-	CzcD
NODE 17	62,178	NZ_JALGQY010000018	-	IS3, IS5, IS21, IS481, ISNCY transposase superfamilies; XerC integrase	TauE
NODE 29	44,217	NZ_JALGQY010000029	-	LGT_TIGR03299 phage/plasmid-like protein; COG5377 phage protein superfamily; AlpA superfamily; Inovirus_Gp2 superfamily; IS66 transposase family; XerC integrase	Fur; SmtA
NODE 38	27,781	NZ_JALGQY010000038.1	rep_pAB02_ORF2	IS3, IS481, IS6 transposase families; XerC integrase	RcnR-FrmR-like_DUF156; MefA protein family; AcrR; GST_N_GTT1/GST_C superfamily
NODE 39	26,463	NZ_JALGQY010000039	-	IS5_2 transposase family	RND_mfp superfamily OtsA, OtsB
NODE 43	18,602	NZ_JALGQY010000042.1	RepM	IS481 and IS3 transposase families Relaxase; MobC	CopA, CopB
NODE 48	14,852	NZ_JALGQY010000047	-	IS3 transposase family	ArsR/SmtB family; TauE
NODE 55	8994	NZ_JALGQY010000052.1	Rep_3; RepM; rep_pAB02_ORF2;	MobA_MobL; TraA_Ti	TerC superfamily; SUL1 superfamily
NODE 57	8544	NZ_JALGQY010000054	-	IS5_3 transposase family	ArsH, ArsC, ArsO, ArsR, Acr3; cztS_silS_copS Pfam00520
NODE 66	6322	NZ_JALGQY010000055.1	Rep_3; RepM; rep_pAB02_ORF2;	-	RamA
NODE 130	3934	NZ_JALGQY010000063	-	IS6, IS240 transposase family	CadR-PbrR Kup
NODE 211	3202	NZ_JALGQY010000065	-	Phage_antiter_Q	Beta-lactamase; Phenol_MetA_deg
NODE 375	2618	NZ_JALGQY010000069	-	Serine recombinase PinE recombinase	ABC_trans_N
NODE 1680	1426	NZ_JALGQY010000076	-	-	CrcB
NODE 1947	1329	NZ_JALGQY010000077	-	-	CzcD; MerR; CadR-PbrR; ZntA; ZitB
NODE 2568	1144	NZ_JALGQY010000083	-	MobA_MobL; TraA_Ti	TelA

## Data Availability

The data presented in this study are available on request from the corresponding author.

## Supplementary Materials

The following supporting information can be downloaded at: https://www.mdpi.com/article/10.3390/life13081728/s1, Table S1: Comparison of the general genome characteristics of various Acinetobacter strains deposited in the GenBank database using the RAST server; Figure S1: Phylogenetic analysis of the 16S rRNA gene of the isolate *Acinetobacter* sp. K1 using the TYGS server; Table S2: The list of bacterial strains that were used to create the ANI similarity matrix and subsequent phylogenetic analysis; Table S3: ANI similarity matrix of the analyzed strains; Figure S2: UPGMA phylogenetic tree of different strains of *A. lwoffii*, *A. idrijaensis* MII, *A. mesopotamicus* DSM 26953, *A. pecorum*, *Prolinoborus fasciculus* CIP 103579 and *Acinetobacter* sp. K1 constructed based on the similarity matrix constructed from the ANI values between the analyzed strains; Table S4: Prediction of the inclusion of *Acinetobacter* sp. K1 genome contigs into chromosomal or plasmid DNA using the server mlplasmids v2.1.0 in correlation with the average GC content of individual contigs; Table S5: Genetic analysis of individual contig sequences of the *Acinetobacter* sp. K1 genome, which at the nucleotide level show similarities with *Acinetobacter* spp. plasmid DNA or encode genes for plasmid replication proteins, proteins involved in HGT processes or genes involved in various mechanisms of resistance and adaptation. The summary analysis was created by a combination of analyses using the servers, databases and online tools blastn, blastx, RAST, CD-Search (Conserved Domain Database), ORF finder, mlplasmids v2.1.0, Genomics %G~C Content Calculator.
