# Supplementary material for: Genetic Basis of Acinetobacter sp. K1 Adaptation Mechanisms to Extreme Environmental Conditions
Source: Life (Basel). 2023 Aug 11;13(8):1728. doi: 10.3390/life13081728 (PMC10455571; doi:10.3390/life13081728)
Supplement: Supplementary file 1 [file life-13-01728-s001.zip › life-2495056-supplementary.pdf]

Supplementary material

Genetic basis of *Acinetobacter* sp. K1 adaptation mechanisms to extreme environmental conditions

Nikola Petrová 1, Jana Kisková 1, Mariana Kolesárová1 and Peter Pristaš 1,2,*

[1]Department of Microbiology, Institute of Biology and Ecology, Faculty of Sciences, Pavol Jozef Safarik University in Kosice, Srobarova 2, Kosice, 041 54, Slovak Republic

[2]Centre of Biosciences, Institute of Animal Physiology, Slovak Academy of Sciences, Soltesovej 4-6, 040 01 Kosice, Slovakia

[*]Correspondence: peter.pristas@upjs.sk; Tel.: +421 55 234-1201

Table S1. Comparison of the general genome characteristics of various *Acinetobacter* strains deposited in the GenBank database using the RAST server [1-3].

Bacterial strain	GenBank accession No.	Genome size (bp)	Source of isolation Country	Number of contigs	G+C content (%)	Number of coding sequences	Number of RNAs
Acinetobacter sp. K1	NZ_JALGQY000000000.1	3 357 632	brown mud Ziar nad Hronom Slovakia	94	42.8	3 324	89
A. lwoffii ZS207	NZ_CP019143.1	3 259 224	gold mine Poland	1	43.2	3 197	108
A. lwoffii WJ10621	NZ_CM001194.1	3 184 320	clinical isolate China	9	41.6	3 081	106
A. lwoffii CIP7031	NZ_KB849830.1	3 546 109	*Homo sapiens* USA	12	42.8	3 421	90
A. lwoffii UBA2051	DDAT00000000.1	2 880 324	environmental isolate USA	52	43.4	2 681	59
A. lwoffii UBA1649	DCLL00000000.1	3 030 134	environmental isolate USA	63	43.3	2 801	58
A. lwoffii CIP 64.10	NZ_APQS00000000.1	3 206 079	unknown USA	16	43.0	2 992	89
A. lwoffii NBRC109760	NZ_BBSQ00000000.1	3 298 884	unknown Japan	100	43.1	3 130	60
A. lwoffii NIPH478	NZ_APQU00000000.1	3 276 129	ear swab Czech Republic	9	42.9	3 067	79
A. lwoffii NIPH512	NZ_AYHO00000000.1	3 384 618	*Homo sapiens* USA	12	43.1	3 221	96
A. lwoffii NIPH715	NZ_APOT00000000.1	3 454 560	Pus - *Homo sapiens* Czech Republic	26	42.8	3 278	91
A. lwoffii SH145	NZ_ACPN00000000.1	3 481 004	*Homo sapiens* Germany	76	42.6	3 121	68
A. lwoffii TG19636	NZ_AMJG00000000.1	3 497 784	Urine - *Homo sapiens* USA	245	42.8	3 360	84
A. pseudolwoffii	NZ_FLLN00000000.1	3 031 971	unknown	12	43.3	2 832	105

F78			Germany				
A. idrijaensis MII	JQCU00000000.1	3 846 869	mercury mine soil Slovenia:Idrija	689	42.9	4 177	61
A. idrijaensis 3_20180319_Bin_ METABAT_38_1	CAJQPS000000000.1	2 235 053	sea water North Sea	370	43.9	2 500	37
A. idrijaensis WKH.25	JALRKB000000000.1	2 248 042	estuarine open water surface layer South China	743	43.8	2 863	35

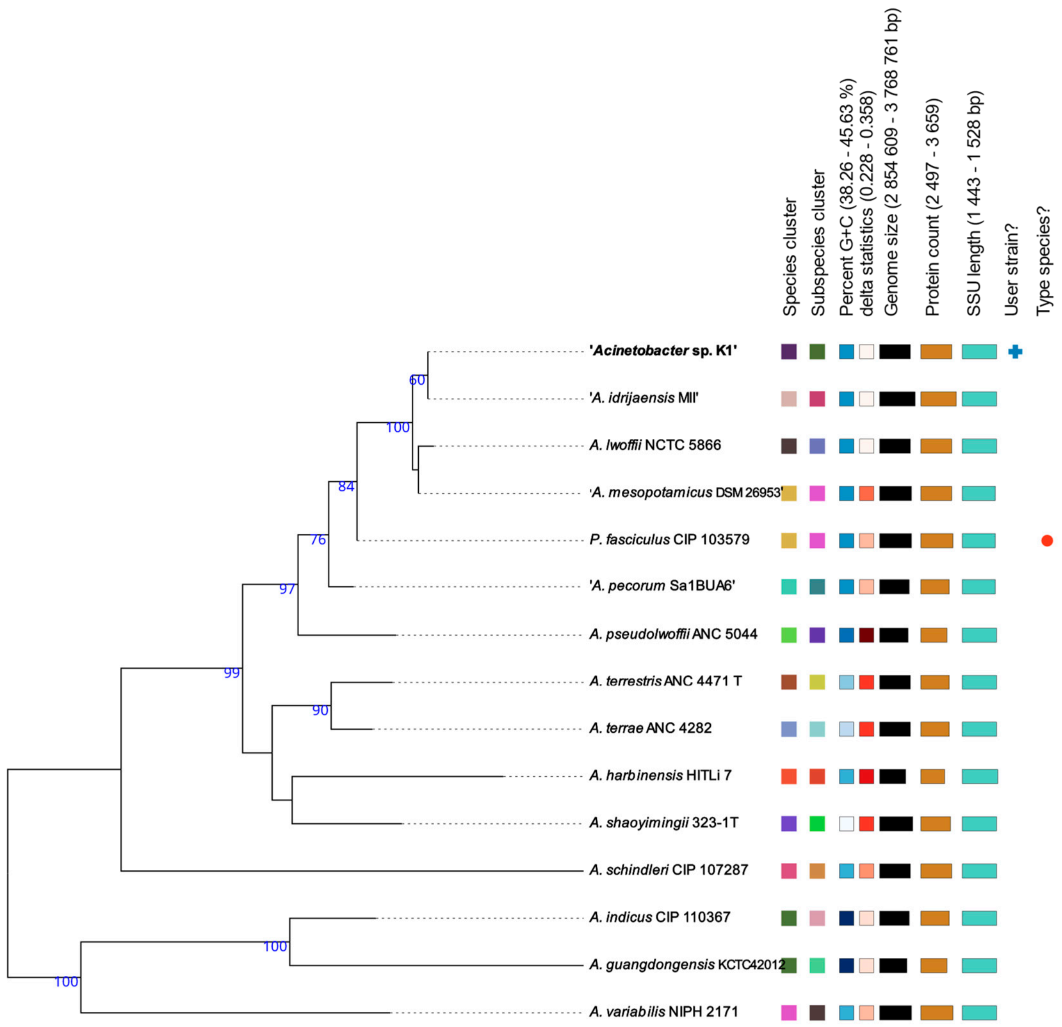

Figure S1. Phylogenetic analysis based on the 16S rDNA gene sequences of *Acinetobacter* spp. using the TYGS server [4]. Tree inferred with FastME 2.1.6.1 [5] from GBDP distances calculated from 16S rDNA gene sequences. The branch lengths are scaled in terms of GBDP distance formula d5. The numbers above branches are GBDP pseudo-bootstrap support values > 60 % from 100 replications, with an average branch support of 78.8 %. The tree was rooted at the midpoint [6]. Leaf labels are annotated by affiliation to species and subspecies clusters, genomic G+C content, delta values, overall genome sequence length, number of proteins and SSU length. Delta statistics permit assessment of accuracy in terms of tree-likeness; the lower the delta value, the greater the accuracy [7].

Table S2. The list of bacterial strains used to create the ANI similarity matrix and phylogenetic analysis.

Bacterial strain	GenBank accession No.	Genome size (bp)	G+C content (%)
Acinetobacter sp. K1	NZ_JALGQY000000000.1	3 357 632	42.76
A. lwoffii NIPH478	NZ_APQU00000000.1	3 301 256	42.88
A. lwoffii UBA2051	DDAT00000000.1	2 885 052	43.35
A. lwoffii SH145	NZ_ACPN00000000.1	3 481 004	42.58
A. lwoffii NIPH715	NZ_APOT00000000.1	3 454 560	42.85
A. lwoffii NBRC109760	NZ_BBSQ00000000.1	3 298 884	43.11
A. lwoffii NIPH512	NZ_AYHO00000000.1	3 384 618	43.09
A. lwoffii NCTC5866**	NZ_AIEL00000000.1	3 353 128	43.04
A. lwoffii CIP7031	NZ_KB849830.1	3 677 680	42.77
A. lwoffii ZS207	NZ_CP019143.1	3 259 224	43.18
A. lwoffii TG19636	NZ_AMJG00000000.1	3 497 784	42.83
A. lwoffii UBA1649	DCLL00000000.1	3 033 116	43.31
A. lwoffii CIP64.10	NZ_APQS00000000.1	3 218 415	43.04
A. lwoffii F78	NZ_FLLN00000000.1	3 031 971	43.32
A. lwoffii WJ10621	NZ_CM001194.1	3 184 320	41.57
A. idrijaensis MII**	GCA_007761495.1	3 846 869	42.90
*A. mesopotamicus DSM 26953/ A. lwoffii GC2***	NZ_JAALFF000000000.1	3 449 117	42.89
*Prolinoborus fasciculus CIP 103579***	GCA_900322255.1	3 450 331	42.99
*A. pecorum Sa1BUA6***	NZ_JACSPT010000000	3 220 949	42.85

*The type strain of *A. lwoffii*; **bacterial strains that were part of the GBDP phylogenetic analysis performed using the TYGS server

Table S3. The ANI similarity matrix of the analyzed strains [8, 9].

	ANI index (%)*																		
	A. lwoffii strain															*Acinetobacter* spp. species			
	**K1**	**NIPH478**	**UBA2051**	**SH145**	**NIPH715**	**NBRC109760**	**NIPH512**	**NCTC5866**	**CIP7031**	**ZS207**	**TG19636**	**UBA1649**	**CIP64.10**	**F78**	**WJ10621**	***A. idrijaensis* MII****	***A. mesopotamicus* DSM 26953****	***P. fasciculus* CIP 103579****	***A. pecorum* Sa1BUA6****
**K1**	-	96.26	96.06	96.00	95.93	95.85	95.85	95.84	95.81	95.76	95.71	95.63	88.64	87.77	76.67	95.94	95.70	95.73	89.82
**NIPH478**	96.26	-	95.94	96.04	96.04	95.82	95.89	95.93	96.08	95.99	95.83	95.78	88.54	87.57	76.87	96.04	95.77	95.91	89.75
**UBA2051**	96.06	95.94	-	95.78	95.84	95.64	95.63	95.63	95.86	95.72	95.64	95.50	88.40	87.17	76.72	95.81	95.58	95.56	89.62
**SH145**	96.00	96.04	95.78	-	96.64	96.15	96.25	96.12	96.92	96.40	96.74	95.91	88.91	88.09	88.09	96.09	96.61	96.55	89.67
**NIPH715**	95.93	96.04	95.84	96.64	-	96.10	96.20	96.22	96.65	96.36	96.74	95.80	89.07	87.92	77.32	95.91	96.56	96.50	89.80
**NBRC109760**	95.85	95.82	95.64	96.15	96.10	-	99.98	99.96	95.95	96.09	96.01	95.55	88.59	87.79	76.79	95.87	95.95	95.93	89.62
**NIPH512**	95.85	95.89	95.63	96.25	96.20	99.98	-	99.95	95.88	96.21	96.14	95.44	88.67	87.83	77.38	95.75	95.89	96.03	89.63
**NCTC5866****	95.84	95.93	95.63	96.12	96.22	99.96	99.95	-	95.90	96.24	96.09	95.57	88.65	87.82	76.95	95.73	95.86	95.96	89.81
**CIP7031**	95,81	96.08	95.86	96.92	96.65	95.95	95.88	95.90	-	96.60	97.07	95.61	89.02	87.96	77.00	95.83	96.96	97.00	89.87
**ZS207**	95.76	95.99	95.72	96.40	96.36	96.09	96.21	96.24	96.60	-	96.09	95.53	88.58	87.57	76.86	96.22	96.73	96.70	89.77
**TG19636**	95.71	95.83	95.64	96.74	96.74	96.01	96.14	96.09	97.07	96.09	-	95.80	88.88	87.96	76.62	95.78	97.03	97.14	89.70
**UBA1649**	95.63	95.78	95.50	95.91	95.80	95.55	95.44	95.57	95.61	95.53	95.80	-	88.71	87.63	76.38	95.83	95.75	95.56	89.45
**CIP64.10**	88.64	88.54	88.40	88.91	89.07	88.59	88.67	88.65	89.02	88.58	88.88	88.71	-	96.60	77.18	88.78	88.87	88.92	86.11
**F78**	87.77	87.57	87.17	88.09	87.92	87.79	87.83	87.82	87.96	87.57	87.96	87.63	96.60	-	76.90	87.79	87.77	87.95	86.08
**WJ10621**	76.67	76.87	76.72	88.09	77.32	76.79	77.38	76.95	77.00	76.86	76.62	76.38	77.18	76.90	-	76.94	77.20	77.29	76.17
A. idrijaensis* MII*	95.94	96.04	95.81	96.09	95.91	95.87	95.75	95.73	95.83	96.22	95.78	95.83	88.78	87.79	76.94	-	95.76	95.83	89.56
A. mesopotamicus* DSM 26953*	95.70	95.77	95.58	96.61	96.56	95.95	95.89	95.86	96.96	96.73	97.03	95.75	88.87	87.77	77.20	95.76	-	97.62	89.72
P. fasciculus* CIP 103579*	95.73	95.91	95.56	96.55	96.50	95.93	96.03	95.96	97.00	96.70	97.14	95.56	88.92	87.95	77.29	95.83	97.62	-	89.75
A. pecorum* Sa1BUA6*	89.82	89.75	89.62	89.67	89.80	89.62	89.63	89.81	89.87	89.77	89.70	89.45	86.11	86.08	76.17	89.56	89.72	89.75	-

*The type strain of *A. lwoffii*; **bacterial strains that were part of the GBDP phylogenetic analysis performed using the TYGS server; *** grey highlighting represents ANI values that are below cut-off level

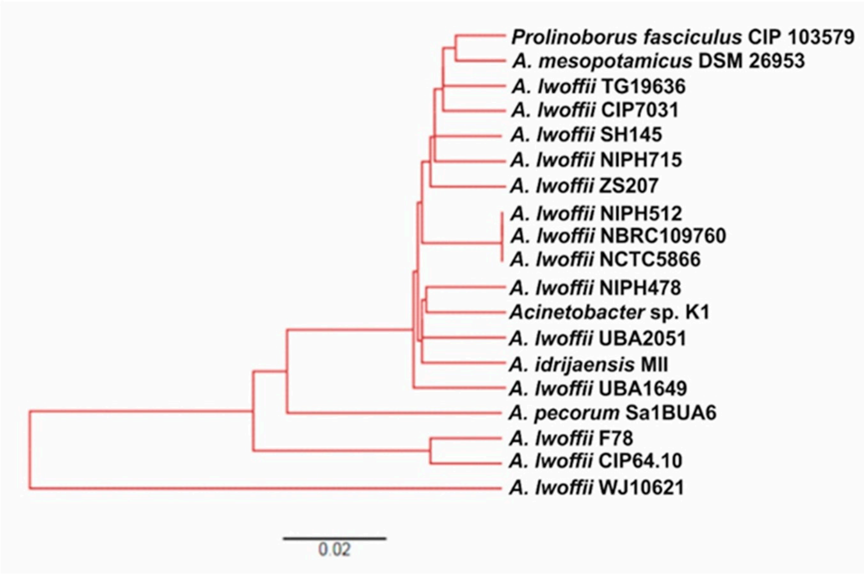

Figure S2. UPGMA phylogenetic tree of different strains of *Acinetobacter* species constructed based on the ANI similarity matrix between the analyzed strains [8-10].

Table S3. Prediction of plasmid and chromosomal DNA in the *Acinetobacter* sp. K1 genome using the server mlplasmids v2.1.0 [11] in correlation with the average GC content of contigs.

Contig	Size (bp)	Probability – chromosome*	Probability - plasmid *	Prediction*	G+C content (%)
NODE 72	5918	0,018	0,982	plasmid	34.6
NODE 55	8994	0,043	0,957	plasmid	34.6
NODE 223	3148	0,043	0,957	plasmid	37.2
NODE 417	2522	0,057	0,943	plasmid	34.8
NODE 228	3051	0,064	0,936	plasmid	37
NODE 68	6259	0,07	0,93	plasmid	32
NODE 130	3934	0,074	0,926	plasmid	38.1
NODE 211	3202	0,114	0,886	plasmid	37.8
NODE 66	6322	0,129	0,871	plasmid	33
NODE 375	2618	0,129	0,871	plasmid	33.3
NODE 2568	1144	0,131	0,869	plasmid	46.8
NODE 1680	1426	0,148	0,852	plasmid	35.4
NODE 2039	1299	0,153	0,847	plasmid	39.8
NODE 110	4254	0,157	0,843	plasmid	33.9
NODE 580	2252	0,159	0,841	plasmid	37.7
NODE 48	14852	0,161	0,839	plasmid	39.5
NODE 97	4628	0,161	0,839	plasmid	34.3
NODE 57	8544	0,176	0,824	plasmid	44.5
NODE 3060	1034	0,176	0,824	plasmid	34.5
NODE 485	2390	0,189	0,811	plasmid	33.6
NODE 42	21048	0,213	0,787	plasmid	35.2
NODE 43	18602	0,237	0,763	plasmid	37.8
NODE 47	15243	0,283	0,717	plasmid	33.6
NODE 1101	1715	0,311	0,689	plasmid	27.9
NODE 161	3534	0,345	0,655	plasmid	42.6
NODE 2715	1110	0,368	0,632	plasmid	39.5
NODE 49	12221	0,375	0,625	plasmid	30.5
NODE 878	1905	0,386	0,614	plasmid	38.3
NODE 29	44217	0,394	0,606	plasmid	43
NODE 13	78976	0,41	0,59	plasmid	43.4
NODE 4-2	34994	0,433	0,567	plasmid	42.8
NODE 31	35965	0,434	0,566	plasmid	44.6
NODE 1947	1329	0,457	0,543	plasmid	37.6
NODE 2408	1188	0,458	0,542	plasmid	42.7
NODE 17	62178	0,465	0,535	plasmid	39.2
NODE 38	27781	0,471	0,529	plasmid	38.6
NODE 39	26463	0,476	0,524	plasmid	42.2
NODE 2098	1282	0,501	0,499	chromosome	37.8
NODE 33	32163	0,519	0,481	chromosome	42.9
NODE 127	3941	0,536	0,464	chromosome	44.2
NODE 100	4556	0,537	0,463	chromosome	43.4

NODE 45	18048	0,547	0,453	chromosome	38.8
NODE 565	2270	0,567	0,433	chromosome	34.5
NODE 6	141043	0,575	0,425	chromosome	43.1
NODE 20	55230	0,58	0,42	chromosome	45.1
NODE 44	18520	0,629	0,371	chromosome	41.7
NODE 2	272913	0,636	0,364	chromosome	43.8
NODE 2641	1126	0,652	0,348	chromosome	48.1
NODE 51	10566	0,656	0,344	chromosome	45.1
NODE 4-1	115960	0,665	0,335	chromosome	43.8
NODE 23	51990	0,673	0,327	chromosome	43.4
NODE 15	68811	0,676	0,324	chromosome	42.6
NODE 8	107513	0,68	0,32	chromosome	42.9
NODE 35	29453	0,719	0,281	chromosome	43.9
NODE 14	73337	0,725	0,275	chromosome	43.9
NODE 5	147787	0,731	0,269	chromosome	45.2
NODE 32	34241	0,746	0,254	chromosome	42.3
NODE 34	31431	0,756	0,244	chromosome	38.7
NODE 27	45448	0,767	0,233	chromosome	42.9
NODE 40	24304	0,77	0,23	chromosome	42.3
NODE 9	102282	0,777	0,223	chromosome	41.3
NODE 16	65633	0,781	0,219	chromosome	42.3
NODE 50	12045	0,794	0,206	chromosome	38.8
NODE 2099	1282	0,794	0,206	chromosome	40
NODE 2138	1270	0,796	0,204	chromosome	48
NODE 19	58134	0,803	0,197	chromosome	43.4
NODE 12	80362	0,818	0,182	chromosome	44.6
NODE 37	27851	0,819	0,181	chromosome	42.6
NODE 10	100485	0,823	0,177	chromosome	43.7
NODE 261	3010	0,831	0,169	chromosome	38.3
NODE 1	316667	0,832	0,168	chromosome	43.7
NODE 21	55014	0,834	0,166	chromosome	42
NODE 22	54990	0,834	0,166	chromosome	41.4
NODE 36	29291	0,835	0,165	chromosome	43.2
NODE 18	61241	0,839	0,161	chromosome	42.9
NODE 3	165452	0,846	0,154	chromosome	43.7
NODE 7	122405	0,856	0,144	chromosome	42.3
NODE 30	43735	0,857	0,143	chromosome	42.6
NODE 67	6294	0,873	0,127	chromosome	43.7
NODE 56	8733	0,889	0,111	chromosome	39.7
NODE 11	93093	0,894	0,106	chromosome	44.6
NODE 46	17232	0,897	0,103	chromosome	41.5
NODE 25	50082	0,922	0,078	chromosome	43.7
NODE 24	50807	0,928	0,072	chromosome	43.3
NODE 28	44964	0,961	0,039	chromosome	41.8
NODE 52	10319	0,97	0,03	chromosome	40.1

Table S4. Genetic analysis of contig sequences of the *Acinetobacter* sp. K1 genome, based on the results of various bioinformatic tools and databases: blastn, blastx [12], RAST [1-3], CD-Search (Conserved Domain Database) [13], ORF finder (https://www.ncbi.nlm.nih.gov/orffinder/), mlplasmids v2.1.0 [11], Genomics %G~C Content Calculator (https://www.sciencebuddies.org/science-fair-projects/references/genomics-g-c-content-calculator).

Plasmid/ NODE	Size (bp)	GenBank accession No.	Replication	HGT	Resistance	Blastn best hit	GenBank accession No.	Coverage Identity (%)	G+C content (%)
pALK1	3 051	ON109139.1	Rep_3; RepM	MobM	-	*A. defluvii* WCHA30; **p6_010030**	CP029394.2	99 91.44	36.97
unnamed1	6 259	NZ_JALGQY010000057.1	rep_pAB02_ORF2; Replikase	Serine recombinase family	-	*A. lwoffii* ED9-5a **chromosome**/ A. pittii 2012N21-164; **p2012N21-164-4**	CP083571.1/ CP033539.1	4 87.44/ 5 78.31	32.03
unnamed2	5 918	NZ_JALGQY010000058.1	Rep_3; RepM	MobA_MobL; TraA_Ti	-	*A. schindleri* HZE23-1, **pHZE23-1-6**	CP044469.1	7 98.07	34.62
NODE 4-2	34 994	NZ_JALGQY010000005	-	IS5_3 transposase superfamily	AcrA, AcrB; RND_mfp superfamily	*Acinetobacter* sp. WY4; **chromosome**	CP053947.1	98 96.96	42.8
NODE 13	78 976	NZ_JALGQY010000014	-	-	CzcD	*A. lwoffii* DSM 2403; **chromosome**	CP118963.1	99 96.56	43.4
NODE 15	68 811	NZ_JALGQY010000016	-	-	CueR; CopA, CopZ;	*A. lwoffii* ED23-35;	CP082143.1	98	42.6

					MerR_1<br>ZntA; MdlB; SUL1<br>ArsR/SmtB family;<br>MFS_YfcJ_like family;	**chromosome**		97.38	
NODE 17	62 178	NZ_JALGQY010000018	-	IS3, IS5, IS21, IS481,<br>ISNCY transposase family;<br>XerC integrase	TauE	*A. haemolyticus* w12; **plasmid p4**	CP085288.1	63<br>97.42	39.2
NODE 29	44 217	NZ_JALGQY010000029	-	LGT_TIGR03299 phage/plasmid-like protein;<br>COG5377 phage protein superfamily;<br>AlpA superfamily;<br>Inovirus_Gp2 superfamily;<br>IS66 transposase family;<br>XerC integrase	Fur; SmtA	*Acinetobacter* sp. WY4;<br>**chromosome**	CP053947.1	82<br>96.87	43
NODE 31	35 965	NZ_JALGQY010000031	-	-	-	*A. lwoffii* FDAARGOS 1394;<br>**chromosome**	CP077369.1	100<br>96.46	44.6
NODE 34	31 431	NZ_JALGQY010000034	-	IS6 transposase family;<br>Serine recombinase	CopA, CopB, CopC, CopD, CopZ;<br>CusS, CusR; PcoB;	*A. seifertii* AS40, **pAS40-2/**<br>*A. johnsonii*	CP061642.1/<br>CP090416.1	63<br>99.56<br>/	38.7

					ChrA, ChrB; CzcD; ZntA; DmeF	ICE_NC **chromosome**		88 95.85	
NODE 38	27 781	NZ_JALGQY010000038.1	rep_pAB02_ORF2	IS3, IS481, IS6 transposase family; XerC integrase	RcnR-FrmR-like_DUF156; MefA protein family; AcrR; GST_N_GTT1/ GST_C superfamily	*A. lwoffii* AL_065, **pAL_065-3**	CP078047.1	94 96.64	38.6
NODE 39	26 463	NZ_JALGQY010000039	-	IS5_2 transposase family	RND_mfp superfamily OtsA, OtsB	*A. lwoffii* VS15; **chromosome**	CP080576.1	98 96.50	42.2
NODE 42	21 048	NZ_JALGQY010000041	-	IS5_3, IS630 superfamily	-	*A. lwoffii* VS15; **chromosome**	CP080576.1	45 97.50	35.2
NODE 43	18 602	NZ_JALGQY010000042.1	RepM	IS481 a IS3 transposase family Relaxase; MobC	CopA, CopB	*A. pseudolwoffii* XMC5X702; **pXMC5X702-tetX-145k**	CP084302.1	68 98.89	37.83
NODE 47	15 243	NZ_JALGQY010000046	repM_Acin; rep_pAB02_ORF2; DNA_BRE_C super family;	Serine recombinase family; VirD4 superfamily; ComFC superfamily; Phage_int_SAM_2 phage integrase superfamily	-	*A. johnsonii* FDAARGOS_1094; **unnamed6**	CP068191.1	30 93.78	33.64
NODE 48	14 852	NZ_JALGQY010000047	-	IS3 transposase	ArsR/SmtB family;	*A. indicus*	CP024620.2	60	39.5

				family	TauE	SGAir0564;<br>**chromosome**		100	
NODE 49	12 221	NZ_JALGQY010000048	-	-	-	*A. johnsonii* AJ312;<br>**chromosome**	CP045051.1	45<br>88.99	30.5
NODE 50	12 045	NZ_JALGQY010000049	-	IS3, IS5_3<br>transposase family	ArsC, ArsO, Acr3;<br>SUL1 superfamily	*A. lwoffii* ED9-5a;<br>**pALWED3.1**	CP083572.1	74<br>99.49	38.8
NODE 55	8 994	NZ_JALGQY010000052.1	Rep_3; RepM;<br>rep_pAB02_O<br>RF2;	MobA_MobL;<br>TraA_Ti	TerC superfamily;<br>SUL1 superfamily	*A. lwoffii* EK30A;<br>**pALWEK1.1**	CP032102.1	79<br>95.17	36.21
NODE 56	8 733	NZ_JALGQY010000053	-	-	CzcA, CzcD;<br>TolC; CadR-PbrR<br>CusB/AcrA	*A. lwoffii*<br>FDAARGOS_552;<br>**unnamed1**	CP046295.1	100<br>99.26	39.7
NODE 57	8 544	NZ_JALGQY010000054	-	IS5_3 transposase<br>family	ArsH, ArsC, ArsO,<br>ArsR, Acr3;<br>cztS_silS_copS<br>Pfam00520	*A. lwoffii* DSM<br>2403;<br>**chromosome**	CP118963.1	99<br>99.06	44.5
NODE 66	6 322	NZ_JALGQY010000055.1	Rep_3; RepM;<br>rep_pAB02_O<br>RF2;	-	RamA	*A. baumannii* SDF;<br>**p3ABSDF**	CU468233.1	52<br>99.08	32.96
NODE 97	4 628	NZ_JALGQY010000059	-	IS5_2 transposase<br>family;<br>ComEC	-	*A. indicus* C15_T;<br>**pC15-2**	CP048656.1	64<br>99.79	34.3

NODE 110	4 254	NZ_JALGQY010000061	-	IS5_3 transposase family	-	*Acinetobacter* sp. WY4; **chromosome**	CP053947.1	98<br>96.87	33.9
NODE 130	3 934	NZ_JALGQY010000063	-	IS6, IS240 transposase family	CadR-PbrR<br>Kup	*A. schindleri* BDT2091; **pBDT2091-4**	CP094838.1	76<br>93.55	38.1
NODE 161	3 534	NZ_JALGQY010000064	-	-	-	*A. lwoffii* H7; **chromosome**	CP072549.1	97<br>98.79	42.6
NODE 211	3 202	NZ_JALGQY010000065	-	Phage_antiter_Q	Beta-lactamase; Phenol_MetA_deg	*A. lwoffii* ED45-23; **pALWED2.8**	CP032123.1	70<br>99.65	37.8
NODE 223	3 148	NZ_JALGQY010000066.1	Rep_3; RepM; rep_pAB02_ORF2;	-	-	*A. lwoffii* AL_065; **pAL_065-5**	CP078049.1	100<br>97.19	37.23
NODE 261	3 010	NZ_JALGQY010000068	-	-	CzcD	*A. lwoffii* FDAARGOS_557; **unnamed3**	CP054804.1	100<br>99.37	38.3
NODE 375	2 618	NZ_JALGQY010000069	-	Serine recombinase PinE rekombinase	ABC_trans_N	*A. lwoffii* FDAARGOS_551; **unnamed1**	CP054821.1	53<br>97.32	33.3
NODE 417	2 522	NZ_JALGQY010000070	-	IS3 transposase family	-	*A. bereziniae* AB839; **pAB839-1**	CP119243.1	56<br>98.41	34.8

NODE 485	2 390	NZ_JALGQY010000071	-	PBECR3 superfamily	-	*A. lwoffii* AL_065; **pAL_065-5**	CP078049.1	37 98.99	33.6
NODE 580	2 252	NZ_JALGQY010000073	-	ISNCY_2 transposase family; mob_myst_B	-	*A. lwoffii* ZS207; **pmZS**	CP019144.2	97 99.3	37.7
NODE 878	1 905	NZ_JALGQY010000074	-	IS5_3 transposase family; INT_Rci_Hp1_C recombinase	-	*A. lwoffii* ZS207; **chromosome**	CP019143.2	87 95.01	38.3
NODE 1101	1 715	NZ_JALGQY010000075	-	-	-	*A. indicus* TQ18; **chromosome**	CP045131.1	17 90.10	27.9
NODE 1680	1 426	NZ_JALGQY010000076	-	-	CrcB	*A. lwoffii* DSM 2403; **unnamed3**	CP118966.1	100 100	35.4
NODE 1947	1 329	NZ_JALGQY010000077	-	-	CzcD; MerR; CadR-PbrR; ZntA; ZitB	*A. lwoffii* DSM 2403; **unnamed1**	CP118964.1	100 99.62	37.6
NODE 2039	1 299	NZ_JALGQY010000078	-	Transposase_mut superfamily; IS1249 transposase family	-	*A. haemolyticus* AN3; **chromosome**/ *A. towneri* AT235; **unnamed**	CP031984.1/ CP062186.1	100 99.77/ 100 99.69	39.8
NODE 2408	1 188	NZ_JALGQY010000082	-	IS3 transposase family	-	*A. baumannii* VB35179;	CP040054.1/ CP024418.1	99 99.92/	42.7

						**unnamed1**/ *A. baumannii* A388; **chromosome**		100 99.92	
NODE 2568	1 144	NZ_JALGQY010000083	-	MobA_MobL; TraA_Ti	TelA	*Acinetobacter* sp. 10FS3-1; **p10FS3-1-9**	CP039152.1	100 98.51	46.8
NODE 2715	1 110	NZ_JALGQY010000085	-	-	-	*A. lwoffii* DSM 2403; **chromosome**	CP118963.1	93 98.94	39.5
NODE 3060	1 034	NZ_JALGQY010000086	-	IS3 transposase family	-	*A. wuhouensis* WCHA60; **chromosome**/ *A. wuhouensis* WCHA60; **p2_010060**	CP031716.1/ CP031710.1	57 98.99/ 57 99.32	34.5

*Contigs showing the discrepancy between different bioinformatic analyses used (orange), contigs having a higher average GC content than most plasmid-related contigs (grey), contigs with the lowest average GC content within the entire genome (blue).

References

1. Aziz, R.K.; Bartels, D.; Best, A.A. et al. The RAST Server: Rapid annotations using subsystems technology. BMC Genom. 2008, 9, 75; https://doi.org/10.1186/1471-2164-9-75
2. Overbeek, R.; Olson, R.; Pusch, G.D. et al. The SEED and the Rapid Annotation of microbial genomes using Subsystems Technology (RAST). Nucleic Acids Res. 2014, 42, 206-214; https://doi.org/10.1093/nar/gkt1226
3. Brettin, T.; Davis, J.J.; Disz, T. et al. RASTtk: A modular and extensible implementation of the RAST algorithm for building custom annotation pipelines and annotating batches of genomes. Sci. Rep. 2015, 5, 8365; https://doi.org/10.1038/srep08365
4. Meier-Kolthoff, J.P.; Göker, M. TYGS is an automated high-throughput platform for state-of-the-art genome-based taxonomy. Nat. Commun. 2019, 10, 2182; https://doi.org/10.1038/s41467-019-10210-3
5. Lefort, V.; Desper, R.; Gascuel, O. FastME 2.0: A comprehensive, accurate, and fast distance-based phylogeny inference program. *Mol. Biol. Evol*. 2015, 32, 2798–2800; https://doi.org/10.1093/molbev/msv150
6. Farris, J.S. Estimating phylogenetic trees from distance matrices. *Am. Nat*. 1972, 106, 645–667; https://www.jstor.org/stable/2459725
7. Holland, B.R.; Huber, K.T.; Dress, A.; Moulton, V. Delta plots: a tool for analyzing phylogenetic distance data. *Mol. Biol. Evol*. 2002, 19, 2051–2059; https://doi.org/10.1093/oxfordjournals.molbev.a004030
8. Goris, J.; Konstantinidis, K.T.; Klappenbach, J.A. et al. DNA–DNA hybridization values and their relationship to whole-genome sequence similarities. Int. J. Syst. Evol. Microbiol. 2007, 57(1), 81-91; https://doi.org/10.1099/ijs.0.64483-0
9. Rodriguez-R, L.M.; Konstantinidis, K.T. Estimating coverage in metagenomic data sets and why it matters. ISME J 2014, 8(11), 2349-2351; https://doi.org/10.1038/ismej.2014.76
10. Moore, R.M.; Harrison, A.O.; Mcallister, S.M. et al. 2020. Iroki: automatic customization and visualization of phylogenetic trees. PeerJ 2020, 8, e8584; https://doi.org/10.7717/peerj.8584
11. Arredondo-Alonso, S.; Rogers, M.R.C.; Braat, J.C. et al. mlplasmids: a user-friendly tool to predict plasmid- and chromosome-derived sequences for single species. Microb. Genom. 2018, 4(1); https://doi.org/10.1099/mgen.0.000224
12. Altschul, S.F.; Gish, W.; Miller, W. et al. Basic local alignment search tool. J. Mol. Biol. 1990, 215, 403–10; https://doi.org/10.1016/S0022-2836(05)80360-2
13. Marchler-Bauer, A.; Lu, S.; Anderson, J.B. et al. CDD: a Conserved Domain Database for the functional annotation of proteins. Nucleic Acids Res. 2011, 39, 225-229; https://doi.org/10.1093/nar/gkq1189
